# Involvement of the pro-oncogenic enzyme fatty acid synthase in the hallmarks of cancer: a promising target in anti-cancer therapies

**DOI:** 10.1038/s41389-023-00460-8

**Published:** 2023-03-18

**Authors:** Dimitri Vanauberg, Céline Schulz, Tony Lefebvre

**Affiliations:** Univ. Lille, CNRS, UMR 8576 - UGSF - Unité de Glycobiologie Structurale et Fonctionnelle, F-59000 Lille, France

**Keywords:** Oncogenes, Biomarkers

## Abstract

An accelerated de novo lipogenesis (DNL) flux is a common characteristic of cancer cells required to sustain a high proliferation rate. The DNL enzyme fatty acid synthase (FASN) is overexpressed in many cancers and is pivotal for the increased production of fatty acids. There is increasing evidences of the involvement of FASN in several hallmarks of cancer linked to its ability to promote cell proliferation via membranes biosynthesis. In this review we discuss about the implication of FASN in the resistance to cell death and in the deregulation of cellular energetics by increasing nucleic acids, protein and lipid synthesis. FASN also promotes cell proliferation, cell invasion, metastasis and angiogenesis by enabling the building of lipid rafts and consequently to the localization of oncogenic receptors such as HER2 and c-Met in membrane microdomains. Finally, FASN is involved in immune escape by repressing the activation of pro-inflammatory cells and promoting the recruitment of M2 macrophages and T regulatory cells in the tumor microenvironment. Here, we provide an overview of the involvement of the pro-oncogenic enzyme in the hallmarks of cancer making FASN a promising target in anti-cancer therapy to circumvent resistance to chemotherapies.

## A long enzyme in a few words

Fatty acid synthase (FASN), previously characterized as OA-519 for Oncogenic Antigen-519 [1], is a ubiquitous and cytosolic enzyme. FASN is the second enzyme of de novo lipogenesis (DNL) catalyzing the synthesis of palmitic acid (C16:0) by using malonyl-CoA and acetyl-CoA as substrates, and NADPH,H^+^, as co-substrate. FASN is functional as a head-to-tail [2] or head-to-head [3] homodimer. Each monomer exhibits seven distinct enzymatic activities working in a sequential and coordinated manner plus an acyl carrier protein (ACP) that carry the acyl group during its elongation [4]. The final product is released by hydrolysis catalyzed by the thioesterase (TE) domain of FASN. The complete structure of mammalian FASN has been solved by X-ray crystallography except for the flexible ACP and TE domains [5]. FASN is not only involved in energy storage in the form of triglycerides but it also actively participates in the synthesis of membrane components and second messengers, and, indirectly, in protein modification by acylation [4]. At the organism level FASN is essential for gestation [6], lactation [7], respiration [8] and ageing [9].

**Fig. 1:**
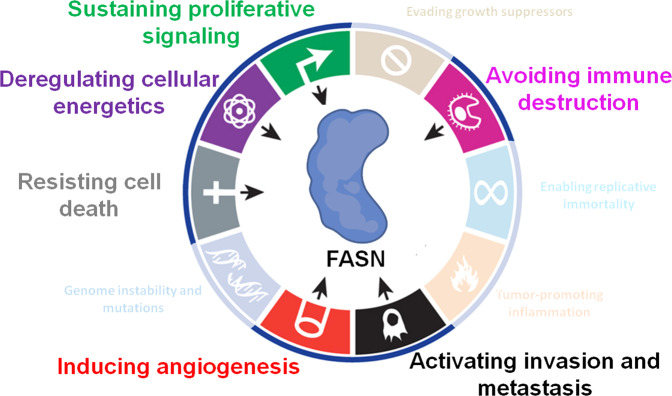
Upregulation of FASN correlates with several hallmarks of cancer. The pro-oncogenic enzyme FASN is involved in sustained proliferation, dysregulation of cell energetics, resistance to cell death, induction of angiogenesis, activation of invasion and metastasis, and prevention of immune destruction. Adapted from Hanahan and Weinberg, 2011 [25].

From a pathological point of view, the implication of FASN in tumorigenesis was first identified in human breast carcinoma cells in 1994 [10]. Thus, FASN is overexpressed in breast cancer [11] but also in many other types of cancers: colorectal [12], prostate [13], stomach [14], esophageal [15], lung [16], pancreatic [17], ovarian [18], hepatic [19, 20], melanoma [21], glioma [22] and in primary effusion lymphoma (PEL) [23]. FASN is often a poor prognostic marker, its overexpression being correlated to a decrease in the survival of patients. However, it is not considered as an oncogene as its overexpression in healthy cells does not induce a malignant transformation as shown for hepatocytes [24]. Through the years, many studies have highlighted the involvement of FASN in different hallmarks of cancer including cell metabolism, proliferation, migration, invasion, resistance to cell death, immune escape, and angiogenesis (Fig. 1): this makes the lipogenic enzyme an interesting and promising target in anti-cancer therapies.

## FASN and the deregulation of cellular energetics

One of the hallmarks of cancer cells is metabolic reprogramming [25]. This includes an increased DNL unlike healthy cells which have a reduced lipogenesis, fatty acid needs being satisfied usually through dietary (exogenous) intake. The acceleration of lipogenesis observed in tumors promotes cell proliferation, survival, invasion and resistance to chemotherapy [26]. The increase in the lipogenic flux is partly related to the Warburg effect and to the overexpression of several metabolic enzymes including FASN. This increase in DNL flux has been not only described in hepatocellular carcinoma (HCC) [26] but also in breast cancer [27], glioblastoma [28], colorectal cancer [29] and in hematologic malignancies like acute myelogenous leukemia and endemic Burkitt lymphoma [30].

**Fig. 2:**
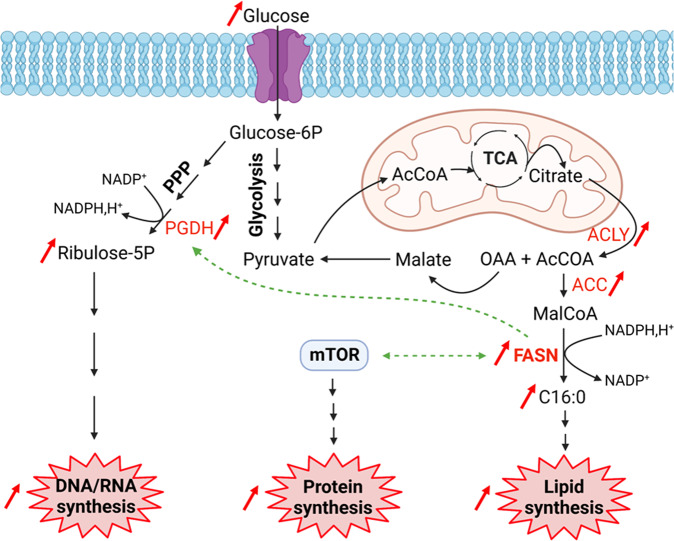
FASN deregulates cell metabolism. Many enzymes are upregulated in cancer cells including the lipogenic enzymes ACLY, ACC, and FASN. Their overexpression leads to an increased lipid synthesis sustained by the Warburg effect. In addition FASN promotes mTOR activation resulting in an increased protein synthesis. FASN also favors the activity of the PPP (pentose-phosphate pathway) enzyme PGDH by increasing the pool of NADP^+^, a co-substrate of the latter. PPP upregulation concomitantly increases DNA/RNA synthesis. The dotted arrows represent an indirect effect. PGDH, 6-phosphogluconate dehydrogenase; AcCoA, acetyl-CoA; MalCoA, malonyl-CoA; TCA, tricarboxylic acid cycle; OAA, oxaloacetic acid; ACLY, ATP citrate lyase; ACC, acetyl-CoA carboxylase; PPP, pentose-phosphate pathway; mTOR, mammalian/mechanistic target of rapamycin; FASN, fatty acid synthase.

The highest levels of the lipogenic enzymes ATP citrate lyase (ACLY), acetyl-CoA carboxylase (ACC), stearoyl-coenzyme A desaturase 1 (SCD1) and FASN have been detected in HCC characterized by an aggressive phenotype [31, 32]. These enzymes are generally overexpressed in HCC explants in association with an increase of triglycerides, fatty acids and cholesterol compared to the adjacent non-tumoral tissue [31]. Recently, it has been shown in non-Hodgkin lymphoma that FASN promoted nucleotide biosynthesis required for supporting malignant proliferation [33]. By using NADPH,H^+^ to produce palmitate, FASN limits the level of the co-substrate and thus promotes 6-phosphogluconate dehydrogenase (PGDH) activity of which NADPH,H^+^ is an allosteric inhibitor. PGDH is the second enzyme of the pentose-phosphate pathway (oxidative branch) that generates ribulose-5-phosphate then converted into ribose-5-phosphate by the keto-isomerase (non-oxidative branch). As a result, FASN indirectly increases DNA/RNA synthesis required for cell proliferation. FASN also promotes protein synthesis through the activation of mammalian/mechanistic target of rapamycin (mTOR) activity in HepG2 [34] and in HCT116 cells [35] (Fig. 2).

## FASN and proliferative signaling

**Fig. 3:**
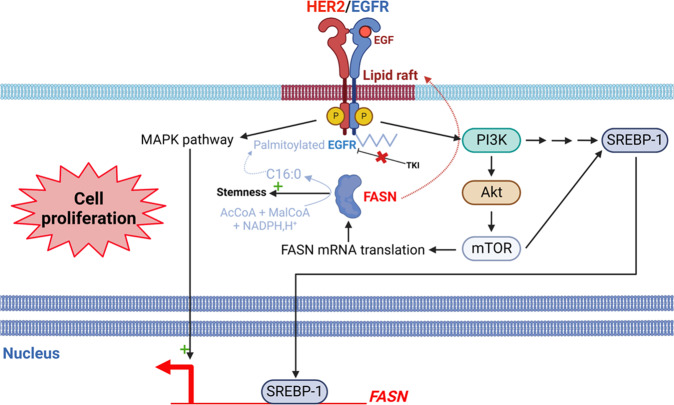
FASN promotes cell proliferation. FASN participates in the elaboration of lipid rafts required for signaling-associated receptors including the oncogenic receptors HER2 and EGFR. Thus, a positive feedback is ON as these receptors drive *FASN* expression via the activation of the MAPK and PI3K/Akt/mTOR pathways. PI3K and mTOR govern the expression of the transcription factor SREBP-1 which in turn induces *FASN* expression. mTOR also regulates FASN mRNA translation. In addition, FASN favors indirectly EGFR palmitoylation which prevents its inhibition by TKI. Finally, FASN promotes stemness and therefore cell proliferation.

Overexpression of FASN in breast cancer cells promotes their survival and proliferation via the hyperactivation of HER1/2 receptors [11]. In turn, HER1/2 induce expression of FASN via activation of the MAPK and PI3K/Akt pathways (Fig. 3) highlighting a positive feedback to maintain high levels of FASN in cancer cells [11]. Jin et al. have demonstrated that inhibition of FASN by C75 (Table 1) in the breast cancer cell lines SKBR3 and BT474 was responsible for an increase in the internalization and degradation of HER2 associated with a decrease in the level of HER2 mRNA [36]. FASN promotes the expression and activation of HER2 probably by allowing its localization in lipid rafts and, conversely, HER2 favors the expression and activity of FASN (Fig. 3). The overexpression of FASN in prostate cancer cells (LNCaP) increases cell proliferation and soft agar growth [13]. In addition, mice bearing xenografts of esophageal squamous cell carcinoma cells (Colo680N) treated with the FASN inhibitor C93 (Table 1) showed a significant inhibition of tumor growth [15]. Together, these observations strongly support a role for FASN in cell proliferation and tumor growth.

**Table 1:** Summary of the different FASN inhibitors discussed in this review.

Compound	Structure	Origin	Targeted domain	Development stage
**C75**		Cerulenin synthetic derivate	β-ketoacyl synthase, thioesterase, enoyl reductase	Pre-clinic
**C93**		Synthetic	β-ketoacyl synthase	Pre-clinic
**EGCG**		Plant-derived polyphenol (natural component of green tea)	β-ketoacyl reductase	Pre-clinic
**G28**		EGCG synthetic derivative	Same as EGCG?	Pre-clinic
**Cerulenin**		Antibiotic produced by *Cephalosporium caerulens*	β-ketoacyl synthase	Pre-clinic
**Curcumin**		Antioxidant produced by *Curcuma longa*	Malonyl/acetyl transferase	Pre-clinic
**Osthole**		Botanical anti-fungal agent produced by *Cnidium monnieri*	?	Pre-clinic
**BI99179**		Synthetic	β-ketoacyl reductase	Pre-clinic
**Orlistat**		Derivative of lipstatin isolated from *Streptomyces toxytricini*	Thioesterase	FDA-approved anti-obesity drug
**TVB-2640**		Imidazopyridine-based inhibitor	β-ketoacyl reductase	In phase II trial
**TVB-3166**		Pre-clinical version of TVB-2640	Same as TVB-2640	Pre-clinic

FASN is overexpressed in breast cancer stem cells (BCSC), promotes stemness, and is hyperactivated in stemness-enriched samples [37]. Its inhibition with the antioxidant epigallocatechin-3-gallate (EGCG) or with the EGCG synthetic derivative G28 (Table 1) diminishes stemness and cell proliferation. It has been recently demonstrated that mTOR promotes FASN expression in HCC cells [34] and in breast cancer cells [38] (Fig. 3). In non-small cell lung cancer (NSCLC) cells with a mutant EGFR, FASN induces indirectly EGFR palmitoylation which prevents its inhibition by tyrosine kinase inhibitors (TKI). This results in a persistent signaling driven by mutated EGFR and consequently to cell proliferation [39] (Fig. 3). DNL is more active in glioblastoma CSCs compared to non-stem cancer cells [40]. FASN is necessary for the expression of markers of glioma stem cells, and for the maintenance of stemness [40]. In this sense, the inhibition of FASN with the natural antibiotic cerulenin (Table 1) blocks cell proliferation and decreases the expression of the glioblastoma stemness markers Nestin, Sox2 and Fatty Acid Binding Protein 7 (FABP7) [40]. On the contrary, cerulenin increases the expression of the differentiation marker glial fibrillary acidic protein (GFAP). At last, FASN is overexpressed in induced pluripotent stem cells, neural stem and progenitor cells and glioma stem-like cells [41]: it is therefore likely that FASN is involved in the maintenance of stemness of CSCs (Fig. 3).

## FASN and resistance to cell death

Migita et al. reported that the expression of FASN in human prostate tumor tissues was statistically significantly inverse to the apoptotic rate [13]. The activation of the intrinsic pathway of apoptosis with camptothecin in the prostate cancer LNCaP cells stably overexpressing FASN did not induce cell death contrary to control LNCaP cells [13] suggesting that FASN blocks the intrinsic pathway of apoptosis. In the same report, the authors showed that transgenic mice overexpressing FASN in the prostate harbored a prostatic hyperplasia associated with a weaker apoptotic rate compared to wild-type mice [13]. A study conducted on LNCaP cells demonstrated that knockdown of FASN favored apoptosis as evidenced by the increased-signal of Annexin V-EGFP and staining with propidium iodide. On the contrary, no effect was observed on fibroblasts viability [42].

Inhibition of FASN with C75 in HepG2 cells induces a G2-phase arrest associated with an increase of p53 expression [43]. Similarly, the treatment of PEL cells with C75 triggers apoptosis contrary to primary B cells [23]. The treatment of MDA-MB-231 cells with the natural FASN inhibitor curcumin (Table 1) or silencing FASN decreases the level of the anti-apoptotic protein Bcl-2 and concomitantly increases the pro-apoptotic protein Bax [44]. These observations strengthen the potent ability of FASN to oppose cancer cell apoptosis likely through their dependence upon DNL compared to healthy cells. Nevertheless, future investigations are necessary to decipher and understand the precise mechanism linking FASN to the extrinsic and intrinsic pathways of apoptosis at the molecular level.

## FASN and cell invasion/metastasis

**Fig. 4:**
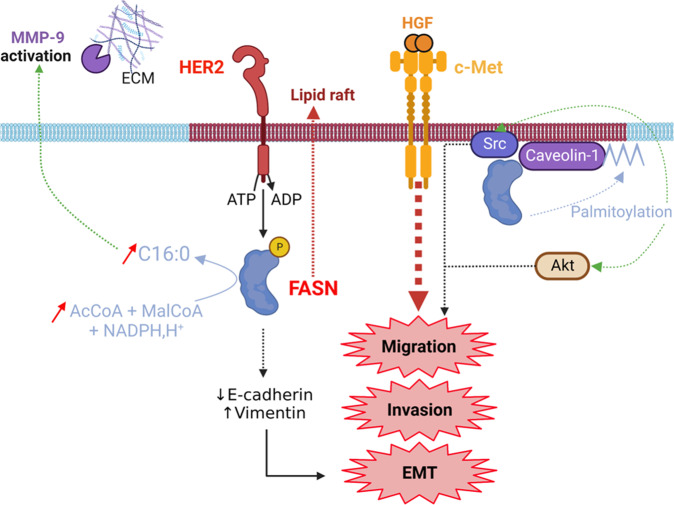
FASN promotes cell migration and invasion. FASN is phosphorylated by HER2 increasing its activity and being responsible for an activation of MMP-9 that degrades the extracellular matrix (ECM). FASN exerts a positive feedback loop by allowing HER2 localization into lipid rafts. Through a respective reduction and increase of E-cadherin and vimentin expression, FASN promotes EMT reinforced by the proper localization of the c-Met receptor into lipid rafts. FASN interacts with Src and caveolin-1. FASN indirectly participates in caveolin-1 palmitoylation which in turn promotes Src and Akt activity leading to cell migration. The dotted arrows represent an indirect effect.

Jin et al. demonstrated that FASN was phosphorylated on Tyr66 by HER2 [36]. This phosphorylation activates FASN leading to cell invasion via an increase in the activity of the metalloproteinase MMP-9 (Fig. 4). Furthermore, FASN mediates the epithelial-mesenchymal transition (EMT) of breast cancer cells as its inhibition with cerulenin increases and decreases respectively the expression of E-cadherin and vimentin, and inhibits cell migration [45]. Hung et al. showed that the inhibition of FASN with the natural compound osthole (Table 1) or with C75 induced a loss of the oncogenic receptor c-Met inhibiting consequently EMT, migration and invasion of MCF-7 cells [46]. The deficit of c-Met was prevented by the addition of palmitate in the culture cell medium. Since c-Met is resident in lipid rafts [47] and that in epithelial cancer cells FASN activity is essential for structuring lipid rafts implicated in cell signaling and migration [48], this study suggests that the loss of FASN prevents c-Met localization in lipid microdomains (Fig. 4). It would be interesting to challenge the correct localization mechanism of c-Met at the level of lipid rafts in near future studies. Also, at the level of these membrane microdomains, FASN has been shown to interact with Src and Caveolin-1. Palmitoylation of caveolin-1, through the indirect activity of FASN, promotes the activation of Src and Akt which increases the migration of prostate cancer cells [49] (Fig. 4).

Expression of FASN in the high metastatic MHCC97H and SK-Hep-1 cell lines is increased compared with low metastatic HCC cell lines [19]. Transfection of HCC SK-Hep-1 cells with shFASN inhibited proliferation, migration and invasion [19]. Supplementation with palmitic acid improves HCC cell migration and activates the TGF-β and Wnt signaling pathways, both responsible for EMT program [50]. The DNL pathway promotes metastasis in mice; inhibiting FASN with the brain-permeable inhibitor BI99179 (Table 1) decreases the growth of brain metastases but does not impact the breast primary tumor [51]. In vivo, inhibiting the transcription factors sterol regulatory element binding proteins (SREBPs), that drive *FASN* expression, by fatostatin blocks both prostate tumor growth and distant metastasis [52]. Finally, cerulenin blocks cell migration and invasion of glioblastoma CSCs [40]. Consequently, FASN promotes metastasis, making the enzyme a prime target for preventing cancer progression and cancer cells spreading.

## FASN and angiogenesis

**Fig. 5:**
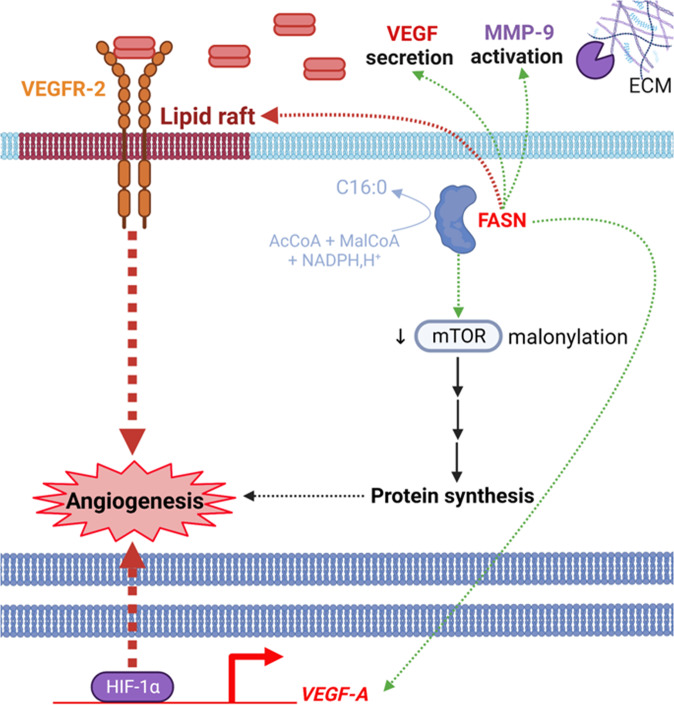
FASN favors angiogenesis. The catalytic activity of FASN probably allows the localization of the oncogenic receptor VEGFR-2 in lipid rafts, a prerequisite to the regulation of angiogenesis. By producing fatty acids, FASN reduces the pool of malonyl-CoA leading to a decrease of mTOR malonylation which promotes its activity and therefore angiogenesis. FASN also controls VEGF secretion and MMP-9 activation to respectively activate VEGFR-2 and degrade the extracellular matrix (ECM), and consequently to sustain angiogenesis. The expression and activity of the transcription factor HIF-1α are positively regulated by FASN; this induces *VEGF-A* expression. The dotted arrows represent an indirect effect.

FASN interferes with angiogenesis. The incubation of breast cancer cells with cerulenin results in a decrease expression of vascular endothelial growth factor (VEGF) and VEGFR-2 [45]. Moreover, FASN promotes the localization of VEGFR-2 on the endothelial cell surface [53]. It can be assumed that FASN promotes the localization of VEGFR-2 at the level of lipid rafts, and thus its activation by VEGF. but, to our knowledge this has never been clearly demonstrated (Fig. 5). In addition, Bruning et al. showed that knockdown of FASN in endothelial cells increased the malonylation of the kinase mTOR due to the excess of malonyl-CoA not used by FASN [54]. Malonylation inhibits mTOR resulting in a decrease in protein synthesis and to a loss of endothelial cell proliferation and angiogenesis (Fig. 5). Interestingly, the inhibition of FASN with orlistat (Table 1), a FDA-approved anti-obesity drug, or with cerulenin, significantly reduces metastases and angiogenesis in a mouse syngeneic model of melanoma [55]. In this model, FASN inhibition induced an increased secretion of an anti-angiogenic isoform of VEGF-A by melanoma cancer cells. Later, Zhou and collaborators showed that the expression level of FASN correlated with microvessels density in human gliomas [22]. They observed in mice orthotopically xenografted with GSCs that silencing FASN using shRNAs or inhibition with C75 decreased microvessels density and tumor growth. Mechanistically, the inhibition of FASN blocked hypoxia-inducible factor-1α (HIF-1α)/VEGF-A signaling and upregulated the anti-angiogenic isoform-VEGF-165b. But, the precise link between FASN and HIF-1α/VEGF-A signaling pathway deserves to be pushed a little further. Zaytseva et al. also demonstrated that the stable knockdown of FASN in colorectal cancer cells induced a decrease of angiogenesis [56]. The culture of human lung microvascular endothelial cells in a medium conditioned from FASN knockdown colorectal cancer cells decreased VEGFR-2 activation, cell proliferation, migration and tubulogenesis. These cancer cells secreted less MMP-9 and pro-angiogenic VEGF (VEGF-189) and conversely secreted more anti-angiogenic VEGF (VEGF-165b).

## FASN and immune escape

**Fig. 6:**
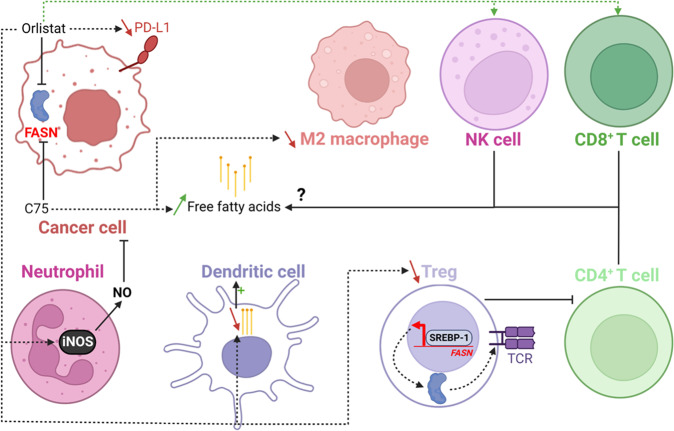
FASN participates to immune escape. Inhibition of FASN by orlistat induces NK cells and CD8^+^ T cells activation. Orlistat decreases the amount of T-regs whose suppressive activity is in part induced by FASN. Orlistat decreases PD-L1 expression, induces the secretion of nitric oxide (NO) by neutrophils and decreases the pool of fatty acids in dendritic cells which increases their maturation. The inhibition of FASN by C75 indirectly decreases the number of M2 macrophages but increases the pool of extracellular fatty acids that probably attract NK and CD4^+^/CD8^+^ T cells in the tumor microenvironnement. FASN is therefore involved in immune escape. The dotted arrows represent an indirect effect.

At last, several studies demonstrated a potential role of FASN in immune escape (Fig. 6). For instance, Sun et al. recently showed that the combined inhibition of FASN with C75 and of phosphatidylinositol 3-kinase-α (PI3Kα) with CYH33 synergistically enhances anti-tumoral immunity [57]. Following the injection of murine breast cancer cells 4T1 into the flank of immunocompetent BALB/c mice treated with C75 and CYH33, an increased apoptotic rate associated to a decrease in cancer cells proliferation was noticed. These observations were correlated to a respective increase and decrease of tumor infiltration by CD4^+^/CD8^+^ T lymphocytes and M2 macrophages. The combined inhibition of FASN and PI3Kα synergistically increased free fatty acids level in the tumor microenvironment. It is tempting to speculate that this increase could be partly responsible for the change observed in the composition in immune cells (Fig. 6).

Another study focusing on regulatory T cells (T-regs) highlighted the involvement of FASN in immune suppression and therefore in immune escape [58]. Lim and collaborators showed that the activity of SREBPs was upregulated in intratumoral T-regs and promoted tumor growth and PD-1 expression. As *FASN* expression is promoted by SREBP-1, they engineered FASN-deficient T-regs cells and observed that these cells were less mature resulting in a decrease of tumor growth. The addition of palmitate in the medium of FASN-deficient T-regs cells restored the suppressive function of T-regs cells. These authors concluded that FASN signaling contributes to the functional maturation of T-regs cells indirectly via the activation of T cell antigen receptors (TCRs) [58] (Fig. 6).

Moreover, the treatment of a melanoma mouse model (B16-F10 cells/C57BL/6 mice) with orlistat reduced lymph node metastases reinforcing the involvement of FASN in immune escape [59]. Mechanistically, FASN inhibition increases the maturation of intratumoral dendritic cells, stimulates the expression of cytotoxicity markers of CD8^+^ T lymphocytes and natural killer cells and reduces the number of T-regs cells. In addition, blood neutrophils produce more nitric oxide which has antineoplastic effects (Fig. 6). Conversely, lipids produced by ovarian cancer cells overexpressing FASN accumulate in dendritic cells [60]. Dendritic cells are then unable to present the antigens to T lymphocytes and to induce an anti-tumor immune response. Finally, Cioccoloni et al. demonstrated that orlistat impairs the expression of PD-L1 implicated in immune suppression in a T-cell leukemia line [61]. This observation suggests that FASN promotes PD-L1 expression and, as a result, immune escape (Fig. 6).

## General conclusion and perspectives

Due to its pivotal involvement in many features of cancer, FASN is potentially a good anti-cancer therapeutic target. To date, TVB-2640 (Table 1) is the most advanced FASN inhibitor currently used in phase 2 trial. Combined to taxane, TVB-2640 is promising in the treatment of advanced cancers [62]. This first-in-class inhibitor has been tested with patients carrying astrocytoma, NSCLC, breast cancer, peritoneal carcinoma and ovarian cancer [63]. TVB-3166 (Table 1), a smaller analogue of TVB-2640 used in vitro, leads to an endoplasmic *reticulum* stress which prevents the translation of ERα mRNAs in tamoxifen-resistant MCF-7 cells and results in a decrease of tumor growth of xenografted tumors in mice [64].

Of particular interest, PROteolysis TArgeting Chimeric molecules, or PROTACs, are promising tools that could be used to develop next-generation therapeutics to target FASN. PROTACs consist of chimeric molecules that bridge any protein to an E3 ligase so as to induce its proteasomal degradation. There are currently several PROTACs in clinical trials for the treatment of cancer such as ARV-471 (breast cancer) expected to enter phase 3 soon or ARV-110 (prostate cancer) which is in phase 2 [65]. These PROTACs are also combined to other drugs like Everolimus for ARV-471 (NCT05501769) or Abiraterone for ARV-110 (NCT05177042) in some clinical trials in an attempt to improve the treatment of patients. Thus, multi-compound therapies represent promising tools for the management of hard-to-cure cancer patients and could apply to FASN in conjunction with inhibitors currently in clinical phase.

FASN may also be responsible for the resistance to trastuzumab (Herceptin™) in HER2 overexpressing breast cancers. As discussed previously, there is a cross-talk between FASN and HER2 in which each promotes the expression of the other [36] (Figs. 3 and 4). The combination of trastuzumab with FASN blockade presents a synergistic effect on SK-BR-3 breast cancer cells growth inhibition and apoptosis [66]. Moreover, the inhibition of FASN-driven lipid rafts building also negatively affects EGFR-HER2 cross-talk which is of particular importance for trastuzumab resistance [48]. It has been also shown that tamoxifen promotes FASN expression in ER^+^/HER2^+^ breast cancer cells and increases cell proliferation [67]. Thus, FASN could also induce tamoxifen resistance by promoting cell growth. In NSCLC cells with mutated EGFR and resistant to TKI, the inhibition of FASN with orlistat induces EGFR K48-ubiquitination resulting in a reduction of tumor growth associated with an increase in apoptosis in mice xenografted tumors [39]. It would be particularly interesting to check whether this K48-ubiquitination of EGFR actually induces its proteasomal degradation.

FASN inhibition could be used as an adjuvant anti-cancer therapy to those already existing. For example, a synergistic effect of FASN and Thymidylate Synthase respectively inhibited by cerulenin and 5-fluorouracil is observed on cell viability in breast cancer cells [68]. In the same way, FASN blockade induces a synergistic chemo-sensitization of breast cancer cells to microtubule interfering agents such as docetaxel [69], paclitaxel [70] and vinorelbine [71]. As discussed previously the combined inhibition of FASN and PI3Kα synergistically inhibits tumor growth in a murine allograft model by enhancing anti-tumoral immunity [57]. As well, the inhibition of FASN in patients harboring a resistance to c-Met TKI is a promising therapy [46]. Finally, the FASN inhibitor G28 allows to overcome EGFR TKIs resistance in NSCLC [72]. The potential final approval of TVB-2640 or other anti-FASN drugs could therefore offer novel therapies for patients with cancer in an era of widespread resistance to existing drugs.

## Data Availability

All data presented in the current review are publicly available, and all findings summarized here come from articles cited in the reference list and available in the MEDLINE database.
